# Changing trends in total knee replacement

**DOI:** 10.1007/s00590-017-1934-8

**Published:** 2017-03-09

**Authors:** Ewan B. Goudie, Cal Robinson, Phil Walmsley, Ivan Brenkel

**Affiliations:** Victoria Hospital, Hayfield Road, Kirkcaldy, KY2 5AH UK

**Keywords:** Osteoarthritis, Total knee replacement, Demographics, BMI

## Abstract

**Introduction:**

This study evaluates a possible change in the demographics and surgical practice observed in a large cohort of patients undergoing total knee replacement (TKR).

**Patients and methods:**

We performed a retrospective analysis of a prospectively collected data on two groups of consecutive patients undergoing primary TKR. Group one consisted of patients who underwent surgery between 1994 and 1998. Group two consisted of patients who had surgery between 2009 and 2012.

**Results:**

The mean age of group two was significantly greater than that of group one: 68.9 years (68.1–69.7 years) for group one versus 70.1 years (69.6–70.6 years) for group two (*p* = 0.009). The mean BMI of group two was significantly greater than that of group one: 29.5 kg/m^2^ (29.0–29.9 kg/m^2^) for group one versus 32.0 kg/m^2^ (31.7–32.3 kg/m^2^) for group two (*p* < 0.001). The mean pain component of the AKSS was significantly worse in group one than in group two: 28.6 (27.2–30.0) for group one versus 35.5 (34.6–36.4) for group two (*p* < 0.001). The mean function component of the AKSS was significantly worse in group one than in group two: 48.6 (47.3–49.9) for group one versus 51.5 (50.7–52.3) for group two (*p* < 0.001).

**Conclusion:**

This study describes the change in demographics of patients undergoing TKR in our institution over the last two decades.

## Introduction

Osteoarthritis (OA) is a very common disease of the joints and a leading cause of pain and disability in middle-aged and elderly patients [1]. The incidence of knee OA is rising as a result of longer life expectancy and increasing BMI in the population. From 1991 to 2006, the rates of total knee replacement (TKR) more than tripled in women (from 42.5 to 138.7 per 100,000 person-years) and men (from 28.7 to 99.4 per 100,000 person-years) [2].

Whilst the increasing need for TKR has been recognised, it is important to understand how the demographics of the population at risk have altered and surgical practice has evolved over this period. This study aims to describe the changing demographics and surgical practice observed in a large cohort of patients undergoing TKR.

## Patients and methods

Since 1994, all patients undergoing TKR in our institution have been prospectively entered into a database. From this database, we selected patients undergoing TKR for OA of the knee. We used two 4-year time periods set 10 years apart to look for any changes. We performed a retrospective analysis of the prospectively collected data on the two groups of consecutive patients undergoing primary TKR in our unit. Group one consisted of patients who underwent surgery between December 1994 and August 1998 and group two consisted of patients who had surgery between January 2009 and November 2012. Patients in group one were operated on by one of six orthopaedic surgeons who had a general practice. During group two, the unit had expanded to 11 orthopaedic surgeons of which six were performing TKR. Our inclusion criteria were all patients who underwent elective primary unilateral or simultaneous bilateral TKR for arthritis during these periods. We excluded any patient from out of our catchment area, patients referred out of our area, unicompartmental replacement and revision arthroplasty.

All patients underwent assessment prior to surgery and had demographic data including gender, age, height, weight, body mass index (BMI) and American Knee Society Score (AKSS) recorded. Patients were categorised by age (<60 ‘young’, 60–79 ‘standard’ and 80 or older ‘old’) and by BMI to further facilitate comparison between groups. BMI is a measure of body fat based on height and weight. The World Health Organisation (WHO) has classified BMI into six categories: ‘underweight’ (<18.50 kg/m^2^), ‘normal’ (18.50–24.99 kg/m^2^), ‘overweight’ (25.00–29.99 kg/m^2^), ‘Obese Class I’ (30.00–34.99 kg/m^2^), ‘Obese Class II’ (35.00–39.99 kg/m^2^) and ‘Obese Class III—morbidly obese’ (>40.00 kg/m^2^). Peri-operative data including tourniquet time, use of lateral release, haemoglobin drop, requirement for blood transfusion, and length of inpatient hospital stay were recorded. All assessments were conducted by a dedicated research nurse.

Patients with partially complete data were included in the analysis for the demographic or outcome measures for which they had available data. For the purpose of analysis, individuals undergoing simultaneous bilateral TKR were counted as either ‘one patient’ or ‘two knees’ depending on the demographic or outcome variable measured.

All patients underwent a cemented TKR. A tourniquet was used in all cases and let down at the end of the procedure. No drains were used. All patients had a cemented, fixed bearing cruciate retaining knee replacement. Posterior stabilised knee replacements were reserved for use in severe deformities only. Group one had a press fit condylar knee (PFC, DePuy Orthopaedics, Inc., Warsaw, IN). Group two had a PFC sigma knee (PFC Sigma, DePuy Orthopaedics, Inc., Warsaw, IN). The decision to perform a lateral release was made on an individual basis by the operating surgeon. In 1998, we introduced a transfusion protocol. Only patients with a haemoglobin of <8.5 g/dl were transfused or if the patient was symptomatic between 8.5 and 10 g/dl. Group one received 5000 U daltaparin daily as venous thromboprophylaxis starting 12 h post-operatively and was continued for 2 weeks. In 2009, this was changed to rivaroxaban orally 10 mg starting 8–10 h post-operatively. These patients also received tranexamic acid 500 mg just before the tourniquet was released. All cases received prophylaxis for two weeks unless they were high risk when they received it for 5 weeks.

Statistical analysis was performed using IBM SPSS version 19.0. Data were compared with independent sample t tests for parametric data and Chi-square tests for non parametric data. Statistical significance was set at *p* < 0.05.

## Results

A total of 1879 patients underwent 1982 TKRs during the study period. A total of 1776 of the procedures were unilateral TKRs and 103 were simultaneous bilateral TKRs. In group one, there were 544 patients who underwent 627 TKRs between 1994 and 1998. In group two, there were 1335 patients who underwent 1355 TKRs between 2009 and 2012. The number of cases with complete data for each demographic or outcome measure is detailed below.

### Gender

All 1879 patients had complete data for gender. In group one, there were 251 males (46.1%) and 293 females (53.9%). In group two, there were 612 males (45.8%) and 723 females (54.2%). There was no significant difference in the proportion of males to females between group one and group two (*p* = 0.907).

### Age

**Fig. 1:**
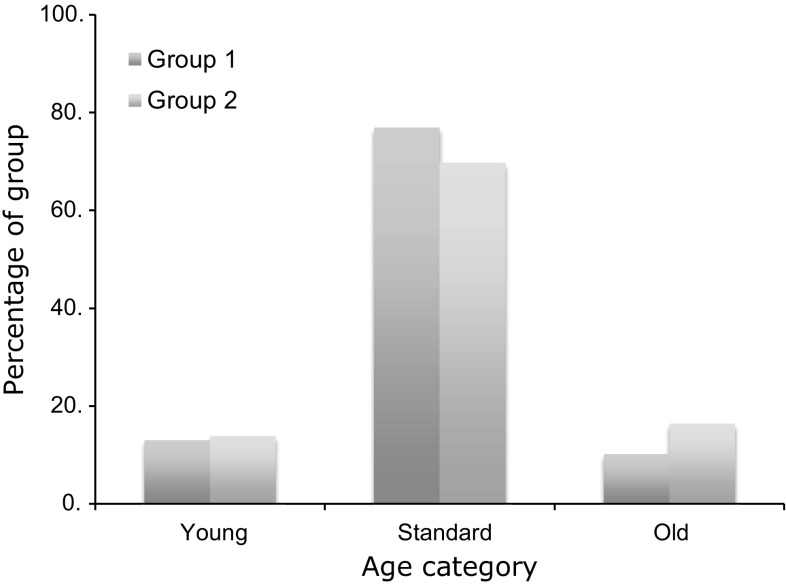
Percentage of patients in each age category in group one and group two

In group one, there were 540 patients (99.3%) with complete data for age. In group two, all 1335 (100%) had complete data for age. The mean age at the time of surgery was significantly greater in group two: 68.9 years (68.1–69.7 years) for group one versus 70.1 years (69.6–70.6 years) for group two (*p* = 0.009). The majority of patients undergoing TKR in both groups were ‘standard’ age (60–79 years). In group one, 415 out of 540 (76.9%) were ‘standard’ age. In group two, 930 out of 1335 (71.2%) were ‘standard’ age. The proportion of patients in each age category was different between the two groups (*p* = 0.001). There were proportionally more ‘standard’ age patients in group one and more ‘young’ (<60 years) and ‘old’ (80 years and older) patients in group two (70 ‘young’: 415 ‘standard’: 55 ‘old’ for group one, 186 ‘young’: 930 ‘standard’: 219 ‘old’ for group 2) (Fig. 1).

### Height, weight and BMI

In group one, there were 427 (78.5%) patients with complete data for height, weight and BMI. In group two, there were 1324 (99.2%) patients with complete data for height, weight and BMI. The mean weight of patients was significantly greater in group two than in group one: 78.3 kg (77.0–79.7 kg) for group one versus 84.9 kg (84.0–85.8 kg) for group two (*p* < 0.001). There was no significant difference in the mean height between the two groups: 163.2 cm (162.3–164.1. cm) for group one versus 162.7 cm (162.2–163.3 cm) for group two (*p* = 0.338). The mean BMI of group two was significantly greater than that of group one: 29.5 kg/m^2^ (29.0–29.9 kg/m^2^) for group one versus 32.0 kg/m^2^ (31.7–32.3 kg/m^2^) for group two (*p* < 0.001).

**Fig. 2:**
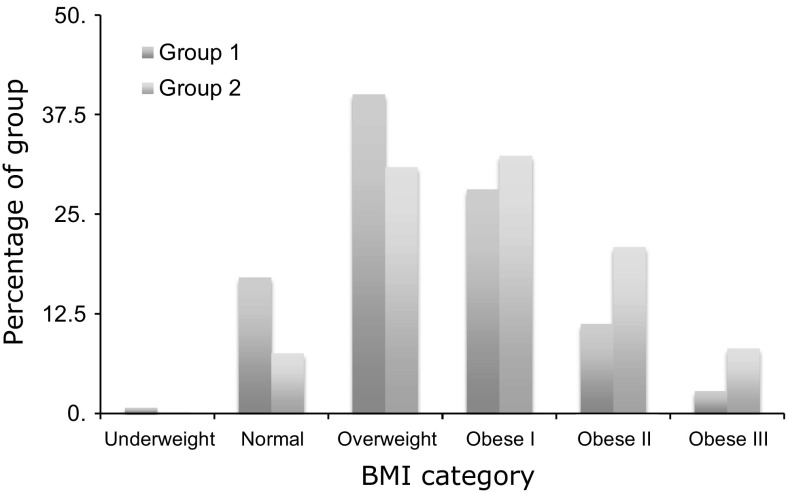
Proportion of patients in each BMI category in group one and group two

The proportion of obese patients (BMI 30 or more) was significantly greater in group two than in group one: 180 out of 427 (42.2%) for group one versus 813 out of 1324 (61.4%) for group two (*p* < 0.001). The proportion of patients in each BMI category was different between group one and group two, with a greater proportion of patients in group two having higher BMIs (*p* < 0.001) (Fig. 2).

### American Knee Society Score (AKSS)

In group one, there were 625 (99.7%) knees with complete data for the pain component of the AKSS. In group two, there were 1351 (99.7%) knees with complete data for the pain component of the AKSS. The mean pain component of the AKSS was significantly worse in group one than in group two: 28.6 (27.2–30.0) for group one versus 35.5 (34.6–36.4) for group two (*p* < 0.001). In group one, there were 621 (99.0%) knees with complete data for the function component of the AKSS. In group two, all 1355 (100%) knees had complete data for the function component of the AKSS. The mean function component of the AKSS was significantly worse in group one than in group two: 48.6 (47.3–49.9) for group one versus 51.5 (50.7–52.3) for group two (*p* < 0.001).

### Tourniquet time

In group one, 594 (94.7%) knees had complete data for tourniquet time. In group two, 1337 (98.7%) had complete data for tourniquet time. The mean tourniquet time was significantly greater for group one than for group two: 71.7 min (70.0–73.1 min) for group one versus 69.2 min (68.3–70.1 min) for group two (*p* = 0.006).

### Lateral release

In group one, 620 (98.9%) knees had complete data for lateral release. In group two, 1295 (95.6%) knees had complete data for lateral release. A lateral release was used in proportionally more knees in group one than in group two: 169 out of 620 knees (27.3%) in group one versus 84 out of 1295 (6.5%) in group two. (*p* < 0.001).

### Simultaneous bilateral surgery

All patients had complete data for unilateral or simultaneous bilateral surgery. A significantly higher proportion of patients underwent simultaneous bilateral TKR in group one compared to group two: 83 out of 544 (15.3%) in group one versus 20 out of 1335 (1.5%) in group two (*p* < 0.001).

### Haemoglobin drop

In group one, 511 (93.9%) patients had complete data for haemoglobin drop. In group two, 1322 (99.0%) patients had complete data for haemoglobin drop. Overall, the mean haemoglobin drop was greater in group one than in group two: 2.46 g/dL (2.36–2.54 g/dL) for group one versus 1.99 g.dL (1.90–2.08 g/dL) for group two (*p* < 0.001). When patients undergoing simultaneous bilateral TKR were excluded, the mean haemoglobin drop remained greater in group one than in group two: 2.31 g/dL (2.23–2.41 g/dL) for group one versus 1.97 g/dL (1.88–2.06 g/dL) for group two. (*p* < 0.001).

### Blood transfusion

In group one, 529 (97.2%) patients had complete data for blood transfusion. In group two, 1330 (99.6%) patients had complete data for blood transfusion. Overall, the proportion of patients receiving post-operative blood transfusion was significantly greater in group one: 189 out of 529 (35.7%) in group one versus 58 out of 1330 (4.4%) in group two (*p* < 0.001). When patients undergoing simultaneous bilateral TKR were excluded, the proportion of patients receiving post-operative blood transfusion remained significantly greater in group one: 137 out of 450 (30.4%) in group one versus 54 out of 1313 (4.1%) in group two (*p* < 0.001).

### Length of stay

In group one, 530 (97.4%) patients had complete data for length of stay. In group two, 1326 (99.3%) patients had complete data for length of stay. The mean length of stay was significantly greater for group one than for group two: 12.2 days (11.5–12.5 days) for group one versus 5.6 days (5.4–5.7 days) for group two (*p* < 0.001). When patients undergoing simultaneous bilateral TKR were excluded, the mean length of stay remained greater for group one than for group two: 11.8 days (11.3–12.4 days) for group one versus 5.5 days (5.4–5.7) for group two (*p* < 0.001).

### Summary of results

**Table:** 

	Group 1	Group 2	*p* value
Age (years)	68.8 (68.1–69.5)	70.1 (69.6–70.6)	0.009*
BMI (kg/m^2^)	29.4 (29.0–29.8)	32.0 (31.7–32.3)	<0.001*
AKSS pain	28.6 (27.2–30.0)	35.5 (34.6–36.4)	<0.001*
AKSS function	48.6 (47.3–49.9)	51.5 (50.7–52.3)	<0.001*
Tourniquet time (mins)	71.7 (70.0–73.1)	69.2 (68.3–70.1)	0.006*
Haemoglobin drop (g/dL) (unilateral TKR)	2.31 (2.23–2.41)	1.97 (1.88–2.06)	<0.001*
Percentage of patients receiving blood transfusion (unilateral TKR)	30.4%	4.1%	<0.001*
Length of stay (days) (unilateral TKR)	11.8 (11.3–12.4)	5.5 (5.4–5.7)	<0.001*

Summary of results (* p < 0.05)

## Discussion

This study demonstrates evidence of a change in the demographic of the patients from our catchment population undergoing TKR over the last two decades. Our study retrospectively analysed prospectively collected data on two unique patient groups. In group one, 544 patients underwent 627 TKRs between 1995 and 1998, and in group two, 1335 patients underwent 1355 TKRs between 2009 and 2013.

The mean age at operation was found to be higher in group two, and it is interesting to note that there were significantly greater proportions of both young (<60 years) and old (>80 years) patients in group two. Both the mean BMI and the proportion of patients with a BMI > 30 kg/m^2^ (obese) were found to be significantly greater in group two than group one. The mean pre-operative pain and function components of the AKSS were found to be significantly worse in group one. The mean length of hospital stay was found to be halved in group two. A significantly lower proportion of patients in group two underwent simultaneous bilateral surgery. Fewer lateral releases were performed in group two. Both the mean haemoglobin drop and the proportion of patients receiving post-operative blood transfusion were greater in group one than group two, even after patients undergoing simultaneous bilateral TKR were excluded.

### Age

The mean age of patients has significantly increased. Operating on an older population carries increased risks and in some cases specific technical challenges. Older knee OA patients have been shown to present for TKR at a more advanced disease stage, with greater pre-operative deformity [3, 4]. They are more likely to suffer from significant comorbidities, which can threaten peri-operative management [5–7]. Finally, complication rates and mortality have been reported to increase with advancing age, in both elective orthopaedic and surgical populations [8–10]. Whilst the mean age at the time of surgery has increased, we are also operating on proportionally more ‘young’ patients than previously. The likely longevity and increased functional demands of these patients may increase the likelihood of prosthesis failure and demand for revision operations, potentially a more complicated procedure at an age where the patient may be less well-equipped to tolerate additional surgery [11–16].

### BMI

Mean BMI significantly increased over our study course. Over 60% of our patients are now obese and 8% are morbidly obese. These findings are consistent with those reported by Odum et al., using US data from over 750,000 TKR procedures [17]. They found that TKR utilisation nearly doubled between 2002–2009, with obesity rising from 6 to 20% of the study population over that period. Whilst greater population obesity has contributed to increased TKR utilisation, it cannot fully explain the trend. Losina et al. suggest that the rising figures represent shifting trends in the management of knee OA, combined with expanding indications for TKR use [18]. Technological advances, greater patient awareness and increased disease surveillance in the past decade are all likely to have contributed to these shifting trends.

The relationship between BMI and outcomes after TKR remains controversial, with limited evidence to suggest that a BMI of between 30–40 kg/m^2^ has a significant impact. We have previously reported no significant difference in the rate of peri-operative complications, revision or implant survival between obese and non-obese patients 9 years after TKR [19]. Another study from our unit showed a significant complication rate in morbidly obese patients (BMI > 40 kg/m^2^) undergoing TKR. Since that time, surgeons in our unit have been reluctant to operate on patients with a BMI over 40 kg/m^2^ [20].

### Disease severity

In current practice, patients are undergoing TKR at an earlier stage than they were 15 years ago. Our contemporary patients had less severe pre-operative symptoms as measured by both the pain and functional components of the AKSS. Advances in prosthetic implants and technical proficiency in TKR have driven increased utilisation of the procedure. Over this period, the evidence for safety, long-term functional outcomes and prosthesis survivorship in younger patients has accumulated and confidence in the procedure in younger patients has grown [21].

### Bilateral TKR

There were significantly fewer simultaneous bilateral TKRs in group two. This reflects an increased awareness of inferior outcomes and increased peri-operative complication rates in simultaneous bilateral TKR [22–24]. In the light of this, we have moved towards sequential unilateral operations with adequate recovery time allowed for between interventions.

### Lateral release

There were fewer lateral releases performed in group two. This change is explained by the difference in prosthesis used in the two groups. Group one received the PFC TKR which does not have a sided femoral component, whereas group two received the PFC Sigma which has sided femoral components which a previous study from this unit has shown to reduce the rate of lateral release [25].

### Haemoglobin drop and blood transfusion

Both the mean haemoglobin drop and the rate of post-operative blood transfusion significantly decreased over the course of our study. The overall drop in transfusion rate is partly explained by the reduced number of simultaneous bilateral cases in group two. Looking at unilateral cases, however, there was still a significant drop in the blood transfusion rate from 30.1% in group one to 4.1% in group two. A previous study from this units has demonstrated how the use of a blood transfusion protocol reduced the post-operative transfusion rate and the routine use of tranexamic acid can further reduce this [26, 27].

### Length of stay

The mean length of hospital stay was found to be halved in group two. The cause for this is multifactorial and includes better pre-operative counselling, improved anaesthesia, reduced requirement for post-operative blood transfusion, faster mobilisation and improved access to physiotherapy. Enhanced recovery programs have been shown to have better patient outcomes and be cost-effective [28–30].

### Study limitations

There are potential limitations that exist in the design of this study. Whist data collection from a single orthopaedic union creates potential sampling bias, it is worth noting that our institution is a large district general hospital and the patient load would be comparable with other units within the UK. A further limitation of our study was the need to segregate patients into two unique groups on the basis of operative date. By rigidly defining these groups, we enabled comparative analysis between the two but restricted our ability to precisely define trends over the study period.

### Conclusion

In summary, our study has shown that the prevalence of obesity has significantly increased in patients undergoing TKR. We additionally found that a greater proportion of both older and younger patients were undergoing TKR and that patients are being operated on at a lower threshold of disease severity. These findings are valuable in their prediction of ongoing trends and the implications they hold on the future provision of elective orthopaedic services. We would recommend the future development of a meta-analysis on this subject. We further recommend ongoing epidemiological analysis of regional and national data on TKR utilisation, in order to precisely define current and predict future trends when planning future service provision.
